# Regional adipose distribution and metabolically unhealthy phenotype in Chinese adults: evidence from China National Health Survey

**DOI:** 10.1265/ehpm.24-00154

**Published:** 2025-01-18

**Authors:** Binbin Lin, Yaoda Hu, Huijing He, Xingming Chen, Qiong Ou, Yawen Liu, Tan Xu, Ji Tu, Ang Li, Qihang Liu, Tianshu Xi, Zhiming Lu, Weihao Wang, Haibo Huang, Da Xu, Zhili Chen, Zichao Wang, Guangliang Shan

**Affiliations:** 1 Department of Epidemiology and Statistics, Institute of Basic Medical Sciences Chinese Academy of Medical Sciences, School of Basic Medicine Peking Union Medical College, Beijing, China; 2 State Key Laboratory of Common Mechanism Research for Major Diseases, Beijing, China; 3 Department of Otolaryngology-Head and Neck Surgery, State Key Laboratory of Complex Severe and Rare Diseases, Peking Union Medical College Hospital, Peking Union Medical College and Chinese Academy of Medical Sciences, Beijing, China; 4 Sleep Center, Department of Respiratory and Critical Care Medicine, Guangdong Provincial People’s Hospital (Guangdong Academy of Medical Sciences), Southern Medical University, Guangzhou, China; 5 Department of Epidemiology and Biostatistics, School of Public Health of Jilin University, Changchun, China; 6 Department of Epidemiology, School of Public Health, Jiangsu Key Laboratory of Preventive and Translational Medicine for Geriatric Diseases, MOE Key Laboratory of Geriatric Diseases and Immunology, Suzhou Medical College of Soochow University, Suzhou, China; 7 School of Population Medicine and Public Health, Chinese Academy of Medical Sciences and Peking Union Medical College, Beijing, China

**Keywords:** Metabolic health, Adiposity, Body fat distribution, Body mass index

## Abstract

**Background:**

The mechanisms distinguishing metabolically healthy from unhealthy phenotypes within the same BMI categories remain unclear. This study aimed to investigate the associations between regional fat distribution and metabolically unhealthy phenotypes in Chinese adults across different BMI categories.

**Methods:**

This cross-sectional study involving 11833 Chinese adults aged 20 years and older. Covariance analysis, adjusted for age, compared the percentage of regional fat (trunk, leg, or arm fat divided by whole-body fat) between metabolically healthy and unhealthy participants. Trends in regional fat percentage with the number of metabolic abnormalities were assessed by the Jonckheere-Terpstra test. Odds ratios (ORs) and their 95% confidence intervals (CIs) were estimated by logistic regression models. All analyses were performed separately by sex.

**Results:**

In non-obese individuals, metabolically unhealthy participants exhibited higher percent trunk fat and lower percent leg fat compared to healthy participants. Additionally, percent trunk fat increased and percent leg fat decreased with the number of metabolic abnormalities. After adjustment for demographic and lifestyle factors, as well as BMI, higher percent trunk fat was associated with increased odds of being metabolically unhealthy [highest vs. lowest quartile: ORs (95%CI) of 1.64 (1.35, 2.00) for men and 2.00 (1.63, 2.46) for women]. Conversely, compared with the lowest quartile, the ORs (95%CI) of metabolically unhealthy phenotype in the highest quartile for percent arm and leg fat were 0.64 (0.53, 0.78) and 0.60 (0.49, 0.74) for men, and 0.72 (0.56, 0.93) and 0.46 (0.36, 0.59) for women, respectively. Significant interactions between BMI and percentage of trunk and leg fat were observed in both sexes, with stronger associations found in individuals with normal weight and overweight.

**Conclusions:**

Trunk fat is associated with a higher risk of metabolically unhealthy phenotype, while leg and arm fat are protective factors. Regional fat distribution assessments are crucial for identifying metabolically unhealthy phenotypes, particularly in non-obese individuals.

**Supplementary information:**

The online version contains supplementary material available at https://doi.org/10.1265/ehpm.24-00154.

## Introduction

The prevalence of overweight and obesity has surged in China over the past three decades [1]. Body mass index (BMI) is commonly utilized as a surrogate measure for general obesity, and elevated BMI is strongly associated with various metabolic dysfunctions including dyslipidemia, hyperglycemia, and hypertension, all of which significantly elevate the risk of cardiovascular disease [2]. However, obesity is acknowledged as a heterogeneous condition with varying metabolic risk among individuals with the same weight status.

The exact mechanisms underlying the distinction between metabolically healthy and unhealthy phenotypes remain elusive. Emerging evidence suggests that adipose tissue stored in different body depots may exert varying impacts on metabolic health [3]. Several studies have demonstrated the protective role of leg and the detrimental role of trunk fat in metabolic syndrome. However, these studies primarily involved Western populations [4–6], had small sample size [7–9], or focused on children and adolescents [10, 11]. There is a lack of large-scale evidence from Chinese adults. Furthermore, the relationship between regional fat distribution and metabolic abnormalities across different BMI categories has not been thoroughly explored, and there is still controversy regarding the role of arm fat in metabolic health.

Although dual-energy X-ray absorptiometry (DXA) has been recently utilized for precise assessment of body fat distribution, its costliness and lack of portability make it unsuitable for large-scale epidemiological surveys [12]. Multi-frequency bioelectrical impedance analysis (BIA) has emerged as a convenient method to quantify fat mass in the whole-body and specific regions of interest, such as trunk, legs and arms [13]. Therefore, using large-scale cross-sectional data from Chinese adults, we aimed to comprehensively investigate whether fat stored in various regions, as measured by the BIA—including arms, trunk, and legs—confers differential associations with metabolically unhealthy statuses across BMI categories in the general population.

## Methods

### Study design and population

The current study was based on the China National Health Survey (CNHS), a large-scale population-based cross-sectional study initiated in 2012. Detailed descriptions of the study design and method of the study have been published elsewhere [14]. Briefly, the CNHS recruited individuals aged 20 years and older from the community using a multistage, stratified cluster sampling method to participate in a clinical examination. This examination included questionnaires, anthropometric measurements, blood pressure (BP) measurements, and blood sample collection. Individuals with severe mental or physical illnesses, pregnant or lactating women, and active military personnel were excluded. Ethical approval was obtained from the Ethics Committee of the Institute of Basic Medical Sciences, Chinese Academy of Medical Science, and all participants provided written informed consent.

Our study utilized data from three CHNS survey sites in Guangdong, Jilin, and Jiangsu provinces conducted between April 23 and November 14, 2023. Among 13892 participants (age ≥20 years), those had a history of cancer (n = 301), cardiovascular diseases (n = 1475), and psychological diseases (n = 29), or with missing data for body composition measurements (n = 266) and metabolic risk factors (n = 152) were excluded, leaving 11833 eligible participants.

### Data collections

Using a standardized protocol across all study sites, data collection was conducted by trained staff. Information on sociodemographic characteristics, lifestyle factors, medical history, and family medical history was collected through face-to-face interviews using a standard questionnaire. To ensure data accuracy, questionnaires were checked and verified by inspectors after each completion.

Physical examinations were conducted by well-trained staff using standard procedures. Height was measured to the nearest 0.1 cm without shoes using a stadiometer (Seca-285, Hamburg, Germany). Body weight and body composition were assessed using bioelectrical impedance analysis (TANITA MC-780MA, Tokyo, Japan) with participants shoeless and in light clothing. The subject stepped on the foot electrodes barefoot and stood still until body weight was measured. The subject grasped the hand electrode cables, and gently held on the thumb electrode and the palm electrode, thereby providing contact with a total of eight electrodes. Hands were held approximately 20 degrees away from the body until measurements were completed. The inbuilt software was used to calculate the body composition values. Waist circumference (WC) was measured horizontally at the level of the belly button.

BP was measured three times using an automated electronic sphygmomanometer (OMRON, HEM-907), which has been clinically validated. Systolic and diastolic BP were measured 3 times at one-minute intervals, and the mean value of the three readings was used for analysis. Blood samples were collected in the morning after a minimum 8-hour fast for measurements of fasting blood glucose (FBG), total cholesterol (TC), high-density lipoprotein cholesterol (HDL-C), low-density lipoprotein cholesterol (LDL-C), and triglycerides (TG).

### Definitions

BMI was calculated as weight (kg) divided by height squared (m^2^) and categorized into normal-weight (<24 kg/m^2^), overweight (24 to <28 kg/m^2^), and obesity (≥28 kg/m^2^) according to Chinese criteria [15]. Percentage regional fat measures were calculated for the arms, legs, and trunk as the regional fat mass divided by the whole-body fat mass multiplied 100 (i.e., % arm fat, % leg fat, and % trunk fat). Appendicular skeletal muscle mass index (ASMI) was calculated by adjusting the appendicular skeletal muscle mass (kg) for weight (kg) and multiplying by 100 [16]. Low muscle mass (LMM) was defined as ASMI more than one standard deviation below the sex-specific means for young adults (age 20–40): 33.0% in men and 28.0% in women [17]. Central obesity was defined using WC of ≥90 cm for men and ≥85 cm for women [18].

Metabolically unhealthy was defined according to the ATPIII guidelines as meeting at least two of the following criteria [19]: 1) serum triglyceride level ≥1.7 mmol/L or currently on drug treatment for high triglycerides; 2) high-density lipoprotein (HDL) cholesterol level <1.04 mmol/L in men and <1.29 mmol/L in women; 3) diastolic BP ≥85 mmHg or systolic BP ≥130 mmHg or using antihypertensive medications; and 4) fasting glucose level ≥5.6 mmol/L or using antihyperglycemic medications. The waist circumference criterion was not used because of collinearity with BMI [20]. Cross-classification of BMI categories and metabolic status (healthy/unhealthy) created six groups: metabolically healthy and normal-weight (MHNW), metabolically unhealthy and normal-weight (MUNW), metabolically healthy overweight (MHOW), metabolically unhealthy overweight (MUOW), metabolically healthy obesity (MHO), and metabolically unhealthy obesity (MUO).

Sociodemographic covariates included age (as a continuous variable), region (Guangdong, Jilin, and Jiangsu provinces), area of residence (urban or rural), education level (middle school or below, high school or above), annual income (<30000 RMB, ≥30000 RMB). Lifestyle factors included smoking, alcohol intake and physical activity. Subjects were categorized as current smokers or nonsmokers. Drinking status was classified as current drinkers or nondrinkers. Information about leisure time physical activity in the past year was categorized as follows: 1) 5–7 times/week; 2) 3–4 times/week; 3) 1–2 times/week; 4) less than 3 times/month; 5) never. For the current analyses, categories were consolidated into physically inactivity (never and occasionally) and physical active (≥1–2 times/week).

### Statistical analyses

Basic characteristics of the study population were presented as the mean ± SD or median (IQR) for continuous variables and the number (frequency) for categorical variables according to sex. Comparisons between groups were performed using Student’s t tests or Wilcoxon rank sum tests for continuous variables and χ^2^ tests for categorical variables. Due to the significant in body fat between men and women, all the analysis was performed stratified by sex. The age-adjusted Spearman correlation coefficients were utilized to assess the correlation between percentage regional fat and BMI, as well as between percentage regional fat and cardiometabolic traits. Covariance analysis was employed to compare the percentage of regional fat between metabolic healthy and unhealthy participants, adjusted for age. The *P*-values for trend between regional fat percentage and the number of metabolic abnormalities was calculated with the use of Jonckheere-Terpstra test.

The percentage of regional fat was categorized into sex-specific quartiles. We conducted a logistic regression model to estimate odds ratios (ORs) with 95% confidence intervals (CIs) to identify the association between body fat distribution and metabolically unhealthy, using the lowest quartile as the reference with adjustment for covariates. Model 1 included age, region, residence area, and education level, annual income, smoke history, drink history, exercise frequency. Model 2 further adjusted for BMI. Analyses on females were additionally adjusted for menopausal state. We also conducted a trend test using ordinal categorical variables as continuous variables in the logistic models. We performed a stratified analysis based on age (<60 and ≥60 years), BMI status (<24, 24 to <28, and ≥28 kg/m^2^) groups, central obesity (yes and no) and LMM (yes and no). Multiplicative interaction was calculated by cross-product interaction terms in multivariable logistic regression models. To test the robustness of the results, we performed sensitivity analyses by excluding participants with a previous diagnosis of hypertension, diabetes, and dyslipidemia. Finally, we evaluated the joint association of regional fat percentage and muscle mass with the risk of being metabolically unhealthy. Men and women were subdivided into four groups based on the median values of regional fat mass and the cutoff for LMM.

SAS 9.4 (SAS Institute Inc., Cary, NC, USA.) was used to conduct all analyses. The significance level was set as a 2-sided P value <0.05.

## Results

Basic characteristics of the study population by sex are presented in Table 1. A total of 4556 men and 7277 women were included in the analysis. The mean age was 52.0 ± 12.8 years for men and 50.6 ± 12.1 years for women. Men had greater BMI and % trunk fat, and lower total and regional fat mass, % arm fat, and % leg fat compared to women. The prevalence of overweight, obesity and metabolically unhealthy phenotype was higher in men than in women.

**Table 1:** Basic characteristics of the study population by sex

**Variables**	**Total** **(N = 11833)**	**Men** **(N = 4556)**	**Women** **(N = 7277)**	***P* Value**
Age (years), Mean ± SD	51.1 ± 12.4	52.0 ± 12.8	50.6 ± 12.1	<0.0001
Urban, n (%)	7355 (62.4)	2702 (59.6)	4653 (64.2)	<0.0001
Annual income (CHY), Median (IQR)	36000 (20000–60000)	48000 (27500–84000)	33333 (18000–54000)	<0.0001
Educated to high school or above, n (%)	5810 (49.2)	2399 (52.8)	3411 (47.0)	<0.0001
Having history of smoking, n (%)	2787 (23.6)	2636 (57.9)	151 (2.1)	<0.0001
Having history of drinking, n (%)	3350 (28.3)	2754 (60.5)	596 (8.2)	<0.0001
Physical inactivity, n (%)	7270 (61.5)	2772 (60.9)	4498 (61.8)	0.2930
BMI (kg/m^2^), Mean ± SD	24.3 ± 3.4	24.9 ± 3.4	24.0 ± 3.4	<0.0001
Total fat mass (kg), Mean ± SD	18.2 ± 6.8	15.9 ± 6.5	19.6 ± 6.6	<0.0001
Trunk fat mass (kg), Mean ± SD	10.1 ± 4.1	9.1 ± 3.9	10.7 ± 4.1	<0.0001
Arm fat mass (kg), Mean ± SD	1.5 ± 0.8	1.2 ± 0.5	1.7 ± 0.8	<0.0001
Leg fat mass (kg), Mean ± SD	6.6 ± 2.1	5.6 ± 2.1	7.2 ± 1.8	<0.0001
Percent trunk fat, Mean ± SD	54.5 ± 5.0	56.3 ± 4.9	53.4 ± 4.7	<0.0001
Percent arm fat, Mean ± SD	8.0 ± 1.2	7.4 ± 1.0	8.3 ± 1.2	<0.0001
Percent leg fat, Mean ± SD	37.5 ± 5.2	36.2 ± 4.6	38.2 ± 5.4	<0.0001
Overweight, n (%)	1642 (13.9)	802 (17.6)	840 (11.5)	<0.0001
Obesity, n (%)	4425 (37.4)	1914 (42.0)	2511 (34.5)	<0.0001
SBP (mmHg), Mean ± SD	125.3 ± 18.0	129.6 ± 16.5	122.5 ± 18.4	<0.0001
DBP (mmHg), Mean ± SD	77.8 ± 11.1	81.1 ± 11.0	75.7 ± 10.6	<0.0001
FBG (mmol/L), Mean ± SD	5.7 ± 1.4	5.9 ± 1.7	5.5 ± 1.2	<0.0001
HDL-C (mmol/L), Mean ± SD	1.4 ± 0.4	1.3 ± 0.3	1.5 ± 0.4	<0.0001
TG (mmol/L), Median (IQR)	1.3 (0.9–2.0)	1.5 (1.0–2.3)	1.2 (0.9–1.8)	<0.0001
Metabolically unhealthy, n (%)	5365 (45.3)	2480 (54.4)	2885 (39.6)	<0.0001

Abbreviations: BMI, body mass index; SBP, systolic blood pressure; DBP, diastolic blood pressure; FBG, fasting blood glucose; HDL-C, high-density lipoprotein cholesterol; TG, triglycerides; SD, standard deviation; IQR, interquartile range.

**Fig. 1:**
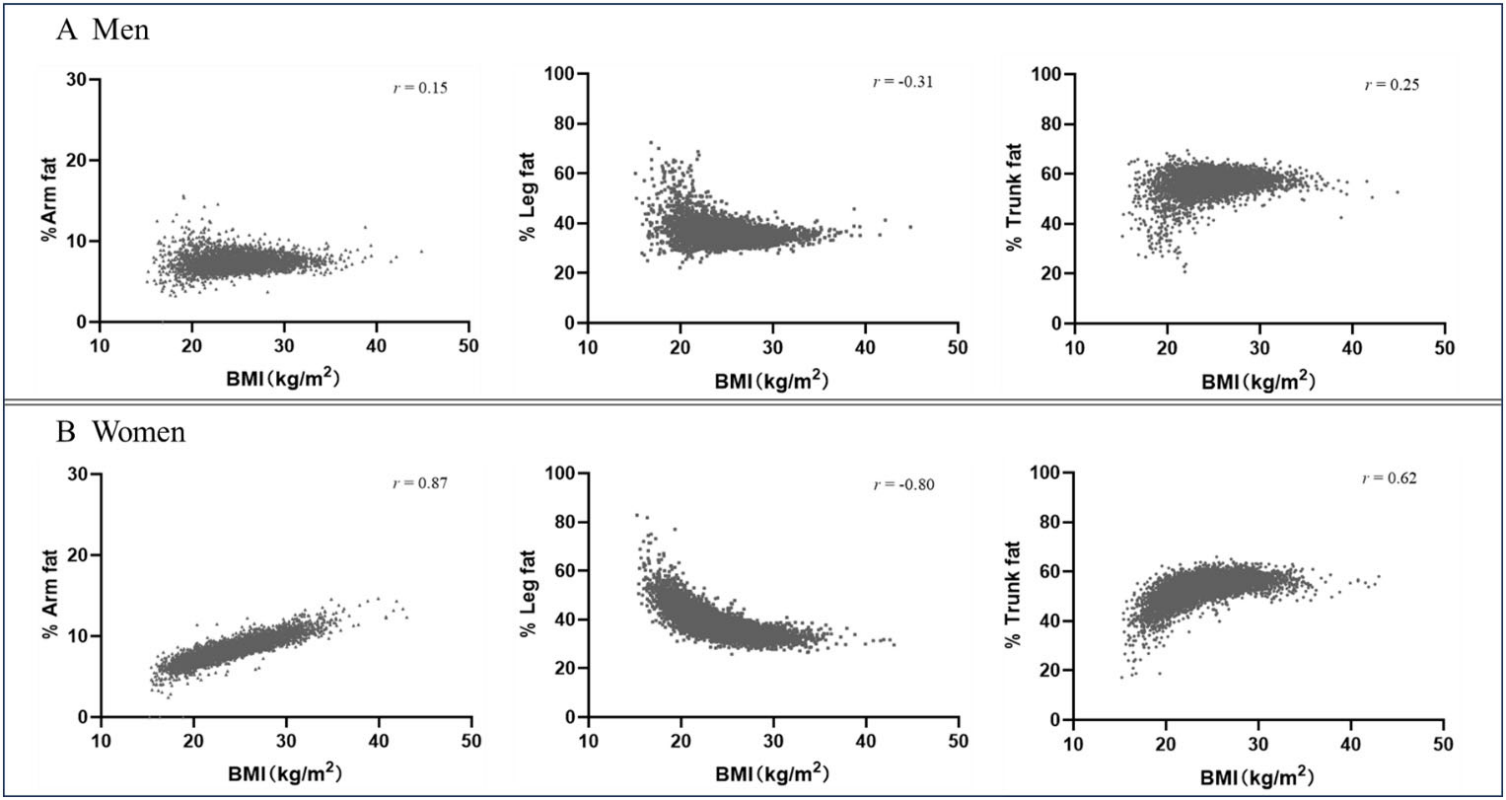
Correlations between regional fat percentage and BMI by sex. Results are Spearman correlation coefficients adjusted for age. P < 0.001 for all correlation estimates. BMI, body mass index.

Figure 1 presents the age-adjusted Spearman correlation coefficients of % arm fat, % leg fat, and % trunk fat with BMI by sex. Both % arm fat and % trunk fat showed strong positive correlations with BMI (*r* > 0.60), whereas % leg fat was inversely correlated with BMI (*r* = −0.80) in women. However, each fat distribution proportion showed weak correlations with BMI in men (absolute *r* < 0.4).

**Fig. 2:**
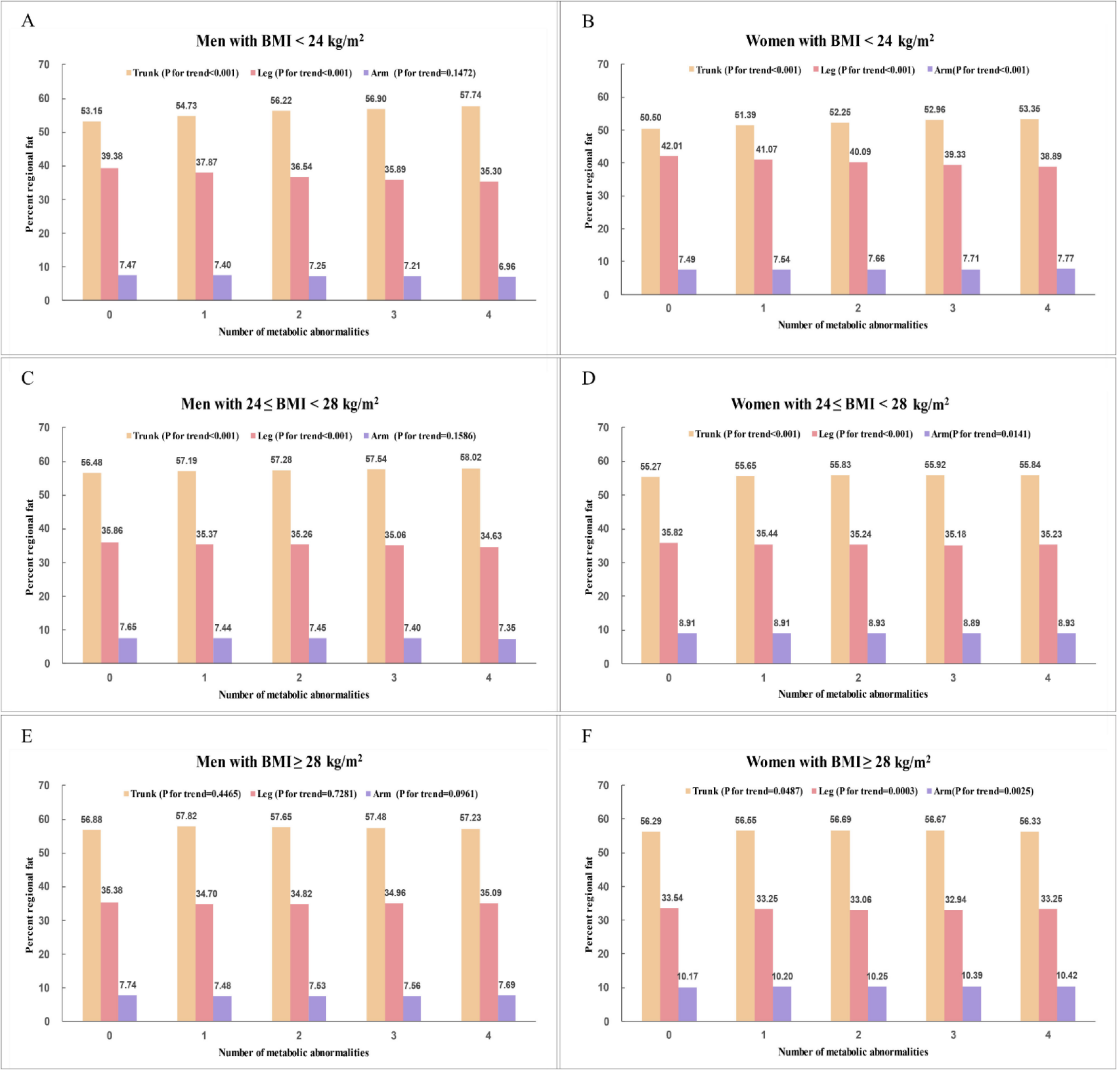
Age-adjusted mean values of regional fat percentage according to the number of metabolic abnormalities. *P*-values for trend were calculated with the use of Jonckheere-Terpstra test. (A) Men with BMI <24 kg/m^2^; (B) Women with BMI <24 kg/m^2^; (C) Men with 24 ≤ BMI < 28 kg/m^2^; (D) Women with 24 ≤ BMI < 28 kg/m^2^; (E) Men with BMI ≥28 kg/m^2^; (F) Women with BMI ≥28 kg/m^2^. BMI, body mass index.

Covariance analysis of the percentage of regional fat between metabolically healthy and unhealthy phenotypes is shown in Table 2. After adjustment for age, metabolically unhealthy participants had significantly lower % leg fat and higher % trunk fat compared to metabolically healthy participants across both sexes in the normal weight and overweight groups. In men with BMI <28 kg/m^2^, % arm fat was lower in metabolically unhealthy participants compared to metabolically healthy participants, while in women with BMI <24 kg/m^2^, % arm fat was higher in metabolically unhealthy participants. However, no significant differences in regional fat percentage were observed between metabolically healthy and unhealthy participants in the obese group. Similarly, significant differences in regional adiposity between metabolically healthy and unhealthy phenotypes were observed in participants without central obesity, but not in those with central obesity (Table S1). Figure 2 illustrates the age-adjusted means of the percentage of regional fat according to the number of metabolic abnormalities. Individuals with a greater number of metabolic abnormalities had higher % trunk fat and lower % leg fat across both sexes in the normal weight and overweight groups (all *P* for trend <0.001). % Arm fat significantly increased with the number of metabolic abnormalities in women across BMI groups, but not in men.

**Table 2:** Covariance analysis of regional fat percentage between metabolically healthy and unhealthy participants.

	**BMI <24 kg/m^2^**			**24 ≤ BMI < 28 kg/m^2^**			**BMI ≥28 kg/m^2^**		
		
	**MHNW**	**MUNW**	***P* Value**	**MHOW**	**MUOW**	***P* Value**	**MHO**	**MUO**	***P* Value**
**Men**									
Percent arm fat	7.43 ± 0.03	7.22 ± 0.05	0.0001	7.51 ± 0.03	7.43 ± 0.02	0.0200	7.54 ± 0.05	7.57 ± 0.03	0.6210
Percent leg fat	38.58 ± 0.17	36.34 ± 0.23	<0.0001	35.52 ± 0.09	35.12 ± 0.07	0.0006	34.87 ± 0.15	34.92 ± 0.08	0.7473
Percent trunk fat	53.99 ± 0.18	56.44 ± 0.24	<0.0001	56.97 ± 0.11	57.45 ± 0.09	0.0009	57.59 ± 0.19	57.51 ± 0.10	0.7057
**Women**									
Percent arm fat	7.51 ± 0.02	7.68 ± 0.03	<0.0001	8.91 ± 0.02	8.92 ± 0.02	0.8712	10.19 ± 0.06	10.33 ± 0.04	0.0438
Percent leg fat	41.62 ± 0.09	39.85 ± 0.16	<0.0001	35.59 ± 0.06	35.22 ± 0.06	<0.0001	33.34 ± 0.12	33.05 ± 0.09	0.0468
Percent trunk fat	50.87 ± 0.09	52.48 ± 0.15	<0.0001	55.49 ± 0.07	55.86 ± 0.07	0.0003	56.47 ± 0.15	56.62 ± 0.11	0.4085

Abbreviations: BMI, body mass index; MHNW, metabolically healthy and normal-weight; MUNW, metabolically unhealthy and normal-weight; MHOW, metabolically healthy overweight; MUOW, metabolically unhealthy overweight; MHO, metabolically healthy obesity; MUO, metabolically unhealthy obesity.

Covariance analysis adjusted for age. Data were shown as least-square mean ± SE (standard error).

Table 3 displays the result of multivariable-adjusted ORs and 95% CIs for metabolically unhealthy status across quartiles of each fat distribution proportion by sex. % Trunk fat was positively associated, whereas % leg fat was inversely associated with metabolically unhealthy status after adjustment for demographic and lifestyle covariate. Further adjustment for BMI yields similar results. The ORs comparing the highest with the lowest quartile were 0.60 (95%CI: 0.49–0.74; *P*-trend: <0.0001) for % leg fat and 1.64 (95%CI: 1.35–2.00; *P*-trend: <0.0001) for % trunk fat in men, and 0.46 (95%CI: 0.36–0.59; *P*-trend: <0.0001) for % leg fat and 2.00 (95%CI: 1.63–2.46; *P*-trend: <0.0001) for % trunk fat in women. Sex differences in the relationship between % arm fat and metabolically unhealthy status were observed with adjustment for demographic and lifestyle covariates (*P*-trend: 0.8300 for men and <0.0001 for women). However, further adjustment for BMI, % arm fat was inversely associated with metabolically unhealthy both in men and women (both *P*-trend <0.0001). Additionally, a significant interaction between sex and % trunk fat as well as % leg fat on the risk of being metabolically unhealthy was observed (*P* for interaction was 0.0010 and 0.0014, respectively). Similar results were observed for ATP-III components (Table S2–S5).

**Table 3:** Association between percent regional fat and metabolically unhealthy by sex

**Variables**	**Quartile 1**	**Quartile 2**	**Quartile 3**	**Quartile 4**	***P*_for trend_**
**Men**					
Percent arm fat					
range	<6.86	6.86 to <7.36	7.36 to <7.91	≥7.91	
Model 1	Reference	1.31 (1.11, 1.56)	1.38 (1.16, 1.63)	0.99 (0.83, 1.19)	0.8300
Model 2	Reference	0.91 (0.75, 1.09)	0.86 (0.71, 1.03)	0.64 (0.53, 0.78)	<0.0001
Percent leg fat					
range	<33.63	33.63 to <35.40	35.40 to <37.67	≥37.67	
Model 1	Reference	1.04 (0.87, 1.25)	0.82 (0.68, 0.98)	0.35 (0.29, 0.43)	<0.0001
Model 2	Reference	0.95 (0.79, 1.15)	0.84 (0.69, 1.02)	0.60 (0.49, 0.74)	<0.0001
Percent trunk fat					
range	<54.55	54.55 to <57.21	57.21 to <59.24	≥59.24	
Model 1	Reference	2.10 (1.77, 2.50)	2.66 (2.23, 3.18)	2.45 (2.03, 2.94)	<0.0001
Model 2	Reference	1.36 (1.13, 1.64)	1.60 (1.32, 1.94)	1.64 (1.35, 2.00)	<0.0001
**Women**					
Percent arm fat					
range	<7.57	7.57 to <8.28	8.28 to <9.06	≥9.06	
Model 1*	Reference	1.80 (1.54, 2.11)	2.33 (1.99, 2.72)	4.21 (3.59, 4.93)	<0.0001
Model 2*	Reference	1.05 (0.88, 1.25)	0.86 (0.71, 1.05)	0.72 (0.56, 0.93)	0.0044
Percent leg fat					
range	<34.56	34.56 to <37.29	37.29 to <40.79	≥40.79	
Model 1*	Reference	0.58 (0.50, 0.66)	0.32 (0.27, 0.37)	0.15 (0.13, 0.18)	<0.0001
Model 2*	Reference	0.84 (0.72, 0.98)	0.64 (0.53, 0.77)	0.46 (0.36, 0.59)	<0.0001
Percent trunk fat					
range	<51.22	51.22 to <54.19	54.19 to <56.60	≥56.60	
Model 1*	Reference	2.04 (1.72, 2.41)	3.56 (3.02, 4.21)	5.02 (4.22, 5.99)	<0.0001
Model 2*	Reference	1.30 (1.09, 1.55)	1.79 (1.49, 2.16)	2.00 (1.63, 2.46)	<0.0001

Data are presented as odds ratio (95% confidence interval).

Model 1: adjusted for age, region, residence area, education level, annual income, smoke history, drink history, exercise frequency.

Model 2: further adjusted for body mass index.

*Models were additionally adjusted for menopausal status.

The *P*_for interaction_ between sex and percent arm fat, percent leg fat, and percent trunk fat were 0.4137, 0.0014, and 0.0010, respectively.

In stratified analysis, the ORs of metabolically unhealthy status related to % regional fat showed no significant differences between age subgroups, except for % leg fat in women (Table S6 and S7). We observed a significant interaction between BMI and % leg fat as well as % trunk fat in both sexes. The associations were not significant in individuals with BMI ≥28 kg/m^2^. The relationship between leg and arm fat and metabolic health differed across the central and non-central obesity groups in women, but not in men. Additionally, we observed a significant modifying effect of LMM on the association between % trunk fat and % leg fat with being metabolically unhealthy in men, and % arm fat with being metabolically unhealthy in women. In sensitivity analysis, similar findings were found when excluding the participants with a previous diagnosis of hypertension, diabetes, and dyslipidemia (Table S8). Additionally, significant correlations between regional fat percentage and cardiometabolic markers were found among these individuals (Table S9).

Compared to the lower % trunk fat and higher LMM group, higher % trunk fat combined with lower LMM was associated with the higher risk of being metabolically unhealthy, but lower % trunk fat and lower LMM were not (Fig. S1). Additionally, participants with lower LMM and lower % limb fat had a particularly higher risk of being metabolically unhealthy compared to those with higher LMM and higher % limb fat.

## Discussion

This large-scale cross-sectional study investigated the associations between regional fat distribution, measured by BIA, and metabolically unhealthy phenotypes in Chinese adults. Our findings revealed contrasting associations between % trunk fat and % limb fat with metabolically unhealthy phenotypes after adjusting for demographic, lifestyle factors, and BMI. Specifically, higher % trunk fat increased the risk of being metabolically unhealthy, while higher % leg fat and % arm fat decreased the risk. Notably, these associations were statistically significant in non-obese participants, not in obese individuals. These findings suggest that fat distribution measured by BIA may provide insights into differential metabolic health in Chinese adults.

Several studies have investigated the impact of leg fat on metabolic health in adults [5, 6, 21]. For instance, a cross-sectional study involving 683 university students aged 18–30 years found that higher levels of leg fat were associated with a better lipid profile [5]. Similarly, Han et al suggested that a lower proportion of fat distributed in the leg region was associated with higher cardiometabolic risk in the Korean population [21]. Additionally, a study of 3220 white and African American adults demonstrated that high leg adiposity was associated with a decreased risk of having two or more cardiometabolic risk factors [6]. However, large-scale studies focusing on Chinese adults are lacking. In our study, we observed a negative association between leg fat mass and a metabolically unhealthy phenotype and its components in both men and women. These findings suggest that fat stored in the legs may play a protective role against metabolic health in Chinese adults.

Consistent with prior research, our findings indicated that higher level of trunk fat was associated with metabolic disturbances, such as insulin resistance, type 2 diabetes, and dyslipidemia [5, 11, 22, 23]. Furthermore, we found that individuals classified as MUOW and MUNW exhibited significantly higher % trunk fat compared to their metabolically healthy counterparts (MHOW and MHNW), respectively. Notably, the detrimental impact of % trunk fat was stronger among individuals with BMI <24 kg/m^2^, irrespective of sex. This suggests that elevated levels of trunk fat may serve as a predictor of metabolic risk, particularly among individuals with normal weight. It’s important to note that trunk fat encompasses both visceral and subcutaneous fat [24]. Previous studies have delineated differences in metabolic function between visceral and subcutaneous fat, with visceral fat showing a stronger association with metabolic risk factors compared to subcutaneous fat [25, 26]. Therefore, the adverse effect of trunk fat may primarily stem from visceral fat accumulation [10].

Previous research on the association between arm fat and metabolic risk has yielded inconsistent findings in adults. A study of 683 adults found no significant effect of arm fat ratio on lipid profile [5]. Yang et al. reported that absolute arm fat was protective against high BP in men but not in women [7]. Another study involving 3220 adults indicated that higher absolute arm adiposity was associated with an increased risk for cardiometabolic risk factors among women, but not men [6]. In our study, we initially observed that % arm fat increased the risk of metabolically unhealthy phenotype in women before adjusting for BMI, but not in men. After adjusting for BMI, % arm fat was associated with a lower risk of metabolically unhealthy phenotype in both genders. These inconsistent results may be due to differences in the form of variables and covariates adjusted for. Unlike studies that adjusted for total body fat or other fat regions—potentially weakening statistical validity due to high correlations—our study employed the relative proportion of regional body fat to whole-body fat, a method that effectively controls for total adiposity [22]. Furthermore, we specifically adjusted for BMI to evaluate the effect of % arm fat on metabolic health at the same BMI level. Our findings suggest that fat stored in the upper limbs may have a protective effect on metabolic health.

The underlying mechanisms for the differential effects of fat stored in different regions on metabolic health remain unclear. Research has shown that regional fat compartments differ in adipose inflammation, lipid storage and turnover, adipokine release, and endocrine effects [27]. Abdominal adipose tissue, particularly visceral fat, is detrimental to metabolic health due to its secretion of proinflammatory cytokines (e.g., tumor necrosis factor-α) and higher lipolytic activity, which elevates free fatty acids levels [26, 28, 29]. Conversely, fat tissue in the legs is primarily stored subcutaneously and is often characterized as a “metabolic sink” due to its lower rate of lipolysis, reduced fatty acid absorption, and secrete more anti-inflammatory cytokines, which provide protective effects against metabolic risk [30].

Our findings have significant clinical practice implications. Although BMI is a cost-effective measure for identifying excessive adipose tissue, it has limitations in distinguishing body composition. We found that higher trunk and lower leg and arm fat are associated with an increased risk of being metabolically unhealthy, especially in non-obese individuals as not classified by BMI. This highlights the importance of fat distribution assessments in addition to BMI for preventing metabolic syndrome. BIA, used for assessing fat distribution, offers advantages in safety, affordability, and portability. Integrating BIA-based fat distribution assessments into routine medical examinations may help identify individuals at heightened risk of metabolic abnormalities, particularly those who are non-obese.

This study has several limitations. First, it is cross-sectional, and therefore, a causal relationship between regional fat depots and metabolic risk factors cannot be inferred. Longitudinal studies are needed to elucidate the long-term impact of specific fat depots on metabolic health in Chinese adults. Secondly, the measurements of body fat relied on the BIA rather than the more accurate DXA method [31]. However, BIA is more affordable, portable, and safe, making it suitable for large-scale population screening. Notably, the multi-frequency BIA used in our study has demonstrated good agreement with DXA in body composition analysis, making it a viable alternative for large epidemiologic studies [32]. For instance, Verney et al. reported a correlation coefficient of 0.852 and a concordance coefficient of 0.844 between DXA-measured and BIA-measured fat mass percentage [33]. Third, we did not collect information on dietary energy intake, which is a significant factor in metabolic health. This omission may introduce potential confounding bias in overestimation or underestimation of body fat on metabolic health.

## Conclusion

Our study revealed the protective role of leg and arm fat, and the detrimental role of trunk fat for metabolic syndrome. The associations of trunk and leg adiposity with metabolic health were stronger in women compared to men. Furthermore, these associations were more pronounced in individuals with normal weight and overweight, but not in those with obesity, underscoring the importance of considering regional fat distribution in identifying metabolically unhealthy phenotypes among non-obese Chinese adults.

## Supplementary information

**Additional file 1:** **Table S1.** Covariance analysis of regional fat percentages between metabolically healthy and unhealthy participants. **Table S2.** Association between percent regional fat and high blood pressure by sex. **Table S3.** Association between percent regional fat and hyperglycemia by sex. **Table S4.** Association between percent regional fat and high triglyceride by sex. **Table S5.** Association between percent regional fat and low HDL-C by sex. **Table S6.** Subgroup analysis of the association between percent regional fat and metabolically unhealthy in men. **Table 7.** Subgroup analysis of the association between percent regional fat and metabolically unhealthy in women. **Table S8.** Sensitivity analyses of percent regional fat and risk of metabolically unhealthy by sex. **Table S9.** Correlation of percent regional fat with cardiometabolic variables by sex. **Figure S1.** Adjusted odds ratios with 95% confidence intervals for metabolically unhealthy by body composition group.

## Declarations

### Availability of data and material

All data analyzed in this paper are available from the corresponding author upon reasonable request.
