# Supplementary material for: Regional adipose distribution and metabolically unhealthy phenotype in Chinese adults: evidence from China National Health Survey
Source: Environ Health Prev Med. 2025 Jan 18;30:5. doi: 10.1265/ehpm.24-00154 (PMC11744028; doi:10.1265/ehpm.24-00154)
Supplement: Supplementary file 1 — Additional file 1: Table S1. Covariance analysis of regional fat percentages between metabolically healthy and unhealthy participants. Table S2. Association between percent regional fat and high blood pressure by sex. Table S3. Association between percent regional fat and hyperglycemia by sex. Table S4. Association between percent regional fat and high triglyceride by sex. Table S5. Association between percent regional fat and low HDL-C by sex. Table S6. Subgroup analysis of the association between percent regional fat and metabolically unhealthy in men. Table 7. Subgroup analysis of the association between percent regional fat and metabolically unhealthy in women. Table S8. Sensitivity analyses of percent regional fat and risk of metabolically unhealthy by sex. Table S9. Correlation of percent regional fat with cardiometabolic variables by sex. Figure S1. Adjusted odds ratios with 95% confidence intervals for metabolically unhealthy by body composition group. [file ehpm-30-005-s001.docx]

**Table S1. Covariance analysis of regional fat percentages between metabolically healthy and unhealthy participants.**

|  | **Non-central obesity** | | |  | **Central obesity** | | |
| --- | --- | --- | --- | --- | --- | --- | --- |
|  | **MH** | **MU** | ***P* Value** |  | **MH** | **MU** | ***P* Value** |
| **Men** |  |  |  |  |  |  |  |
| Percent arm fat | 7.43 ± 0.03 | 7.34 ± 0.04 | 0.0846 |  | 7.42 ± 0.05 | 7.45 ± 0.03 | 0.6323 |
| Percent leg fat | 37.65 ± 0.17 | 36.09 ± 0.19 | <0.0001 |  | 34.39 ± 0.13 | 34.44 ± 0.08 | 0.7291 |
| Percent trunk fat | 54.92 ± 0.18 | 56.57 ± 0.21 | <0.0001 |  | 58.19 ± 0.17 | 58.12 ± 0.10 | 0.6933 |
| **Women** |  |  |  |  |  |  |  |
| Percent arm fat | 7.78 ± 0.02 | 8.08 ± 0.04 | <0.0001 |  | 9.40 ± 0.06 | 9.45 ± 0.04 | 0.5082 |
| Percent leg fat | 40.62 ± 0.12 | 38.55 ± 0.19 | <0.0001 |  | 33.99 ± 0.12 | 33.82 ± 0.08 | 0.2364 |
| Percent trunk fat | 51.60 ± 0.11 | 53.38 ± 0.17 | <0.0001 |  | 56.60 ± 0.13 | 56.73 ± 0.09 | 0.4447 |

Abbreviations: MH, metabolically health; MU, metabolically unhealthy.

Covariance analysis adjusted for age.

Data were shown as least-square mean ± SE (standard error).

Central obesity was defined using waist circumference of ≥90 cm for men and ≥85 cm for women.

**Table S2. Association between percent regional fat and high blood pressure by sex**

| **Variables** | **Quartile 1** | **Quartile 2** | **Quartile 3** | **Quartile 4** | ***P* _for trend_** |
| --- | --- | --- | --- | --- | --- |
| **Men** |  |  |  |  |  |
| Percent arm fat |  |  |  |  |  |
| Model 1 | Reference | 1.13 (0.95, 1.35) | 1.23 (1.03, 1.47) | 1.03 (0.86, 1.24) | 0.5324 |
| Model 2 | Reference | 0.85 (0.70, 1.02) | 0.85 (0.70, 1.03) | 0.74 (0.61, 0.90) | 0.0051 |
| Percent leg fat |  |  |  |  |  |
| Model 1 | Reference | 0.91 (0.75, 1.10) | 0.80 (0.66, 0.97) | 0.46 (0.38, 0.56) | <0.0001 |
| Model 2 | Reference | 0.85 (0.70, 1.03) | 0.82 (0.67, 1.00) | 0.72 (0.58, 0.88) | 0.0023 |
| Percent trunk fat |  |  |  |  |  |
| Model 1 | Reference | 1.64 (1.37, 1.96) | 1.86 (1.55, 2.23) | 1.93 (1.60, 2.34) | <0.0001 |
| Model 2 | Reference | 1.15 (0.96, 1.39) | 1.22 (1.01, 1.49) | 1.39 (1.14, 1.69) | 0.0013 |
| **Women** |  |  |  |  |  |
| Percent arm fat |  |  |  |  |  |
| Model 1^*^ | Reference | 1.41 (1.20, 1.67) | 1.72 (1.46, 2.02) | 3.11 (2.64, 3.66) | <0.0001 |
| Model 2^*^ | Reference | 0.95 (0.79, 1.13) | 0.82 (0.67, 1.01) | 0.83 (0.64, 1.08) | 0.0839 |
| Percent leg fat |  |  |  |  |  |
| Model 1^*^ | Reference | 0.65 (0.56, 0.75) | 0.41 (0.36, 0.48) | 0.28 (0.23, 0.33) | <0.0001 |
| Model 2^*^ | Reference | 0.93 (0.79, 1.09) | 0.79 (0.65, 0.96) | 0.76 (0.59, 0.98) | 0.0140 |
| Percent trunk fat |  |  |  |  |  |
| Model 1^*^ | Reference | 1.52 (1.28, 1.80) | 2.18 (1.84, 2.58) | 2.74 (2.30, 3.27) | <0.0001 |
| Model 2^*^ | Reference | 1.01 (0.84, 1.21) | 1.17 (0.96, 1.41) | 1.18 (0.96, 1.46) | 0.0419 |

High blood pressure was defined as diastolic blood pressure≥85 mmHg or systolic blood pressure≥130 mmHg or using antihypertensive medications.

Data are presented as odds ratio (95% confidence interval).

Model 1: adjusted for age, region, residence area, education level, annual income, smoke history, drink history, and exercise

frequency.

Model 2: further adjusted for body mass index.

^*^ Models were additionally adjusted for menopausal status.

**Table S3. Association between percent regional fat and hyperglycemia by sex**

| **Variables** | **Quartile 1** | **Quartile 2** | **Quartile 3** | **Quartile 4** | ***P* _for trend_** |
| --- | --- | --- | --- | --- | --- |
| **Men** |  |  |  |  |  |
| Percent arm fat |  |  |  |  |  |
| Model 1 | Reference | 1.19 (1.00, 1.43) | 1.30 (1.08, 1.55) | 0.99 (0.82, 1.20) | 0.7950 |
| Model 2 | Reference | 1.00 (0.83, 1.21) | 1.04 (0.87, 1.26) | 0.81 (0.67, 0.99) | 0.0603 |
| Percent leg fat |  |  |  |  |  |
| Model 1 | Reference | 1.10 (0.92, 1.32) | 0.88 (0.73, 1.06) | 0.62 (0.51, 0.75) | <0.0001 |
| Model 2 | Reference | 1.07 (0.89, 1.28) | 0.90 (0.74, 1.08) | 0.81 (0.66, 1.00) | 0.0187 |
| Percent trunk fat |  |  |  |  |  |
| Model 1 | Reference | 1.34 (1.12, 1.61) | 1.59 (1.32, 1.91) | 1.51 (1.25, 1.82) | <0.0001 |
| Model 2 | Reference | 1.08 (0.89, 1.30) | 1.22 (1.01, 1.48) | 1.23 (1.01, 1.49) | 0.0199 |
| **Women** |  |  |  |  |  |
| Percent arm fat |  |  |  |  |  |
| Model 1^*^ | Reference | 1.37 (1.16, 1.62) | 1.54 (1.31, 1.81) | 2.21 (1.88, 2.60) | <0.0001 |
| Model 2^*^ | Reference | 0.97 (0.81, 1.16) | 0.82 (0.67, 1.00) | 0.71 (0.55, 0.92) | 0.0046 |
| Percent leg fat |  |  |  |  |  |
| Model 1^*^ | Reference | 0.64 (0.56, 0.74) | 0.46 (0.39, 0.53) | 0.32 (0.26, 0.38) | <0.0001 |
| Model 2^*^ | Reference | 0.79 (0.68, 0.92) | 0.67 (0.55, 0.81) | 0.58 (0.45, 0.74) | <0.0001 |
| Percent trunk fat |  |  |  |  |  |
| Model 1^*^ | Reference | 1.48 (1.24, 1.76) | 2.03 (1.71, 2.41) | 2.71 (2.27, 3.24) | <0.0001 |
| Model 2^*^ | Reference | 1.13 (0.94, 1.36) | 1.34 (1.11, 1.63) | 1.56 (1.27, 1.93) | <0.0001 |

Hyperglycemia was defined as fasting glucose level≥5.6 mmol/L or using antihyperglycemic medications.

Data are presented as odds ratio (95% confidence interval).

Model 1: adjusted for age, region, residence area, education level, annual income, smoke history, drink history, and exercise

frequency.

Model 2: further adjusted for body mass index.

^*^ Models were additionally adjusted for menopausal status.

**Table S4. Association between percent regional fat and high triglyceride by sex**

| **Variables** | **Quartile 1** | **Quartile 2** | **Quartile 3** | **Quartile 4** | ***P* _for trend_** |
| --- | --- | --- | --- | --- | --- |
| **Men** |  |  |  |  |  |
| Percent arm fat |  |  |  |  |  |
| Model 1 | Reference | 1.21 (1.02, 1.43) | 1.11 (0.93, 1.32) | 0.87 (0.72, 1.04) | 0.0878 |
| Model 2 | Reference | 0.90 (0.75, 1.08) | 0.75 (0.62, 0.90) | 0.60 (0.50, 0.73) | <0.0001 |
| Percent leg fat |  |  |  |  |  |
| Model 1 | Reference | 1.05 (0.88, 1.25) | 0.96 (0.80, 1.15) | 0.41 (0.34, 0.50) | <0.0001 |
| Model 2 | Reference | 0.98 (0.82, 1.18) | 0.99 (0.82, 1.19) | 0.63 (0.51, 0.78) | <0.0001 |
| Percent trunk fat |  |  |  |  |  |
| Model 1 | Reference | 2.10 (1.76, 2.51) | 2.33 (1.95, 2.79) | 2.31 (1.91, 2.79) | <0.0001 |
| Model 2 | Reference | 1.49 (1.24, 1.80) | 1.54 (1.27, 1.87) | 1.68 (1.38, 2.05) | <0.0001 |
| **Women** |  |  |  |  |  |
| Percent arm fat |  |  |  |  |  |
| Model 1^*^ | Reference | 1.75 (1.48, 2.06) | 2.23 (1.90, 2.63) | 2.89 (2.46, 3.40) | <0.0001 |
| Model 2^*^ | Reference | 1.16 (0.97, 1.39) | 1.06 (0.87, 1.30) | 0.76 (0.58, 0.98) | 0.0378 |
| Percent leg fat |  |  |  |  |  |
| Model 1^*^ | Reference | 0.75 (0.65, 0.86) | 0.45 (0.39, 0.53) | 0.20 (0.16, 0.24) | <0.0001 |
| Model 2^*^ | Reference | 0.93 (0.80, 1.09) | 0.67 (0.56, 0.81) | 0.37 (0.29, 0.48) | <0.0001 |
| Percent trunk fat |  |  |  |  |  |
| Model 1^*^ | Reference | 2.20 (1.84, 2.63) | 3.67 (3.08, 4.39) | 4.37 (3.63, 5.26) | <0.0001 |
| Model 2^*^ | Reference | 1.66 (1.37, 2.00) | 2.38 (1.96, 2.89) | 2.44 (1.97, 3.02) | <0.0001 |

High triglyceride was defined as serum triglyceride level≥1.7 mmol/L or currently on drug treatment for high triglycerides.

Data are presented as odds ratio (95% confidence interval).

Model 1: adjusted for age, region, residence area, education level, annual income, smoke history, drink history, and exercise

frequency.

Model 2: further adjusted for body mass index.

^*^ Models were additionally adjusted for menopausal status.

**Table S5. Association between percent regional fat and low HDL-C by sex**

| **Variables** | **Quartile 1** | **Quartile 2** | **Quartile 3** | **Quartile 4** | ***P* _for trend_** |
| --- | --- | --- | --- | --- | --- |
| **Men** |  |  |  |  |  |
| Percent arm fat |  |  |  |  |  |
| Model 1 | Reference | 1.31 (1.08, 1.60) | 1.43 (1.17, 1.75) | 1.23 (1.00, 1.52) | 0.0290 |
| Model 2 | Reference | 1.00 (0.81, 1.23) | 0.99 (0.81, 1.23) | 0.87 (0.70, 1.09) | 0.2573 |
| Percent leg fat |  |  |  |  |  |
| Model 1 | Reference | 0.94 (0.76, 1.15) | 0.99 (0.81, 1.22) | 0.38 (0.30, 0.49) | <0.0001 |
| Model 2 | Reference | 0.87 (0.71, 1.08) | 1.03 (0.83, 1.27) | 0.59 (0.46, 0.76) | 0.0015 |
| Percent trunk fat |  |  |  |  |  |
| Model 1 | Reference | 2.25 (1.83, 2.77) | 2.28 (1.84, 2.81) | 2.18 (1.74, 2.73) | <0.0001 |
| Model 2 | Reference | 1.60 (1.29, 1.99) | 1.52 (1.22, 1.90) | 1.58 (1.25, 2.00) | 0.0012 |
| **Women** |  |  |  |  |  |
| Percent arm fat |  |  |  |  |  |
| Model 1^*^ | Reference | 1.88 (1.59, 2.22) | 2.48 (2.10, 2.92) | 3.77 (3.20, 4.44) | <0.0001 |
| Model 2^*^ | Reference | 1.31 (1.10, 1.56) | 1.30 (1.06, 1.58) | 1.19 (0.92, 1.54) | 0.2462 |
| Percent leg fat |  |  |  |  |  |
| Model 1^*^ | Reference | 0.74 (0.64, 0.86) | 0.44 (0.37, 0.51) | 0.24 (0.20, 0.28) | <0.0001 |
| Model 2^*^ | Reference | 1.03 (0.88, 1.21) | 0.79 (0.65, 0.96) | 0.62 (0.49, 0.80) | <0.0001 |
| Percent trunk fat |  |  |  |  |  |
| Model 1^*^ | Reference | 1.96 (1.67, 2.30) | 2.88 (2.44, 3.40) | 3.70 (3.10, 4.42) | <0.0001 |
| Model 2^*^ | Reference | 1.35 (1.13, 1.60) | 1.63 (1.35, 1.96) | 1.74 (1.41, 2.15) | <0.0001 |

Low HDL-C was defined as HDL-C <1.04 mmol/L in men and <1.29 mmol/L in women.

Data are presented as odds ratio (95% confidence interval).

Model 1: adjusted for age, region, residence area, education level, annual income, smoke history, drink history, and exercise

frequency.

Model 2: further adjusted for body mass index.

^*^ Models were additionally adjusted for menopausal status.

**Table S6. Subgroup analysis of the association between percent regional fat and metabolically unhealthy in men**

| **Variables** | **Quartile 1** | **Quartile 2** | **Quartile 3** | **Quartile 4** | ***P* _for interaction_** |
| --- | --- | --- | --- | --- | --- |
| **Percent arm fat** |  |  |  |  |  |
| Age, years |  |  |  |  | 0.5485 |
| < 60 | Reference | 0.92 (0.74, 1.15) | 0.90 (0.72, 1.13) | 0.64 (0.51, 0.81) |  |
| ≥ 60 | Reference | 0.76 (0.54, 1.06) | 0.82 (0.58, 1.15) | 0.58 (0.41, 0.82) |  |
| BMI, kg/m^2^ |  |  |  |  | 0.5481 |
| < 24 | Reference | 0.89 (0.66, 1.19) | 0.76 (0.57, 1.02) | 0.63 (0.46, 0.86) |  |
| 24 to < 28 | Reference | 0.94 (0.71, 1.24) | 0.92 (0.69, 1.21) | 0.64 (0.48, 0.86) |  |
| ≥ 28 | Reference | 1.07 (0.67, 1.73) | 1.53 (0.92, 2.54) | 0.96 (0.58, 1.59) |  |
| Central obesity |  |  |  |  | 0.8546 |
| No | Reference | 0.79 (0.57, 1.10) | 0.78 (0.55, 1.09) | 0.62 (0.44, 0.89) |  |
| Yes | Reference | 1.10 (0.68, 1.76) | 0.97 (0.61, 1.55) | 0.80 (0.49, 1.32) |  |
| Low muscle mass |  |  |  |  | 0.6512 |
| No | Reference | 0.90 (0.72, 1.13) | 0.96 (0.76, 1.21) | 0.68 (0.53, 0.87) |  |
| Yes | Reference | 1.08 (0.78, 1.51) | 0.97 (0.69, 1.36) | 0.88 (0.59, 1.29) |  |
| **Percent leg fat** |  |  |  |  |  |
| Age, years |  |  |  |  | 0.9331 |
| < 60 | Reference | 0.90 (0.72, 1.13) | 0.77 (0.61, 0.96) | 0.61 (0.48, 0.77) |  |
| ≥ 60 | Reference | 0.90 (0.64, 1.27) | 0.83 (0.59, 1.18) | 0.55 (0.39, 0.79) |  |
| BMI, kg/m^2^ |  |  |  |  | 0.0026 |
| < 24 | Reference | 0.67 (0.50, 0.90) | 0.59 (0.43, 0.80) | 0.51 (0.37, 0.71) |  |
| 24 to < 28 | Reference | 1.01 (0.76, 1.35) | 1.03 (0.76, 1.38) | 0.66 (0.49, 0.89) |  |
| ≥ 28 | Reference | 1.02 (0.62, 1.69) | 1.03 (0.61, 1.74) | 0.95 (0.55, 1.64) |  |
| Central obesity |  |  |  |  | 0.1982 |
| No | Reference | 1.02 (0.73, 1.43) | 0.75 (0.53, 1.07) | 0.60 (0.41, 0.87) |  |
| Yes | Reference | 1.56 (0.96, 2.55) | 1.00 (0.62, 1.64) | 1.01 (0.60, 1.67) |  |
| Low muscle mass |  |  |  |  | 0.0147 |
| No | Reference | 0.87 (0.70, 1.09) | 0.72 (0.58, 0.90) | 0.67 (0.53, 0.84) |  |
| Yes | Reference | 0.97 (0.70, 1.35) | 1.23 (0.86, 1.74) | 1.16 (0.80, 1.69) |  |
| **Percent trunk fat** |  |  |  |  |  |
| Age, years |  |  |  |  | 0.8392 |
| < 60 | Reference | 1.34 (1.07, 1.68) | 1.45 (1.15, 1.83) | 1.66 (1.31, 2.10) |  |
| ≥ 60 | Reference | 1.58 (1.12, 2.22) | 1.71 (1.21, 2.41) | 1.96 (1.37, 2.80) |  |
| BMI, kg/m^2^ |  |  |  |  | 0.0006 |
| < 24 | Reference | 1.08 (0.79, 1.48) | 1.33 (0.97, 1.82) | 1.80 (1.30, 2.48) |  |
| 24 to < 28 | Reference | 1.50 (1.14, 1.97) | 1.77 (1.34, 2.33) | 1.41 (1.06, 1.88) |  |
| ≥ 28 | Reference | 0.94 (0.57, 1.55) | 0.93 (0.55, 1.55) | 0.85 (0.51, 1.44) |  |
| Central obesity |  |  |  |  | 0.4212 |
| No | Reference | 1.43 (1.01, 2.02) | 1.73 (1.21, 2.46) | 1.78 (1.24, 2.56) |  |
| Yes | Reference | 1.15 (0.72, 1.83) | 1.30 (0.80, 2.11) | 1.21 (0.74, 1.99) |  |
| Low muscle mass |  |  |  |  | 0.0372 |
| No | Reference | 1.09 (0.86, 1.38) | 1.37 (1.08, 1.73) | 1.57 (1.24, 1.98) |  |
| Yes | Reference | 1.09 (0.76, 1.54) | 0.86 (0.61, 1.21) | 0.95 (0.66, 1.37) |  |

Data are presented as odds ratio (95% confidence interval).

Models adjusted for age, region, residence area, education level, annual income, smoke history, drink history, and exercise frequency, and BMI.

Abbreviations: BMI, body mass index.

**Table 7. Subgroup analysis of the association between percent regional fat and metabolically unhealthy in women**

| **Variables** | **Quartile 1** | **Quartile 2** | **Quartile 3** | **Quartile 4** | ***P* _for interaction_** |
| --- | --- | --- | --- | --- | --- |
| **Percent arm fat** |  |  |  |  |  |
| Age, years |  |  |  |  | 0.4081 |
| < 60 | Reference | 1.04 (0.85, 1.29) | 0.78 (0.62, 0.99) | 0.69 (0.50, 0.93) |  |
| ≥ 60 | Reference | 1.16 (0.84, 1.60) | 1.03 (0.71, 1.50) | 0.90 (0.55, 1.48) |  |
| BMI, kg/m^2^ |  |  |  |  | 0.1914 |
| < 24 | Reference | 1.08 (0.83, 1.41) | 0.90 (0.68, 1.20) | 0.69 (0.51, 0.94) |  |
| 24 to < 28 | Reference | 0.83 (0.65, 1.06) | 0.78 (0.60, 1.00) | 0.82 (0.62, 1.09) |  |
| ≥ 28 | Reference | 1.23 (0.81, 1.88) | 1.10 (0.71, 1.71) | 1.05 (0.62, 1.80) |  |
| Central obesity |  |  |  |  | 0.0031 |
| No | Reference | 1.32 (0.95, 1.83) | 1.15 (0.80, 1.66) | 0.61 (0.39, 0.94) |  |
| Yes | Reference | 1.04 (0.68, 1.58) | 1.03 (0.65, 1.64) | 0.93 (0.51, 1.70) |  |
| Low muscle mass |  |  |  |  | 0.0076 |
| No | Reference | 1.15 (0.86, 1.55) | 1.10 (0.80, 1.51) | 0.84 (0.57, 1.25) |  |
| Yes | Reference | 1.10 (0.90, 1.36) | 1.00 (0.78, 1.28) | 1.18 (0.84, 1.66) |  |
| **Percent leg fat** |  |  |  |  |  |
| Age, years |  |  |  |  | 0.0059 |
| < 60 | Reference | 0.79 (0.67, 0.95) | 0.57 (0.46, 0.71) | 0.37 (0.28, 0.50) |  |
| ≥ 60 | Reference | 1.28 (0.92, 1.79) | 0.85 (0.60, 1.21) | 0.71 (0.46, 1.10) |  |
| BMI, kg/m^2^ |  |  |  |  | 0.0023 |
| < 24 | Reference | 0.73 (0.59, 0.90) | 0.67 (0.52, 0.86) | 0.52 (0.37, 0.74) |  |
| 24 to < 28 | Reference | 0.94 (0.74, 1.21) | 0.73 (0.56, 0.95) | 0.60 (0.44, 0.81) |  |
| ≥ 28 | Reference | 0.98 (0.63, 1.54) | 1.02 (0.64, 1.64) | 0.84 (0.51, 1.38) |  |
| Central obesity |  |  |  |  | 0.0002 |
| No | Reference | 0.71 (0.54, 0.92) | 0.64 (0.47, 0.88) | 0.34 (0.22, 0.52) |  |
| Yes | Reference | 0.67 (0.44, 1.02) | 0.96 (0.61, 1.49) | 0.65 (0.41, 1.04) |  |
| Low muscle mass |  |  |  |  | 0.1092 |
| No | Reference | 1.04 (0.83, 1.32) | 0.99 (0.76, 1.29) | 0.68 (0.48, 0.97) |  |
| Yes | Reference | 0.97 (0.79, 1.20) | 0.83 (0.66, 1.03) | 0.65 (0.50, 0.83) |  |
| **Percent trunk fat** |  |  |  |  |  |
| Age, years |  |  |  |  | 0.1744 |
| < 60 | Reference | 1.36 (1.10, 1.70) | 1.91 (1.53, 2.39) | 2.15 (1.68, 2.74) |  |
| ≥ 60 | Reference | 1.11 (0.82, 1.50) | 1.45 (1.05, 2.00) | 1.43 (1.01, 2.04) |  |
| BMI, kg/m^2^ |  |  |  |  | 0.0009 |
| < 24 | Reference | 1.32 (0.99, 1.76) | 1.45 (1.07, 1.95) | 2.00 (1.46, 2.76) |  |
| 24 to < 28 | Reference | 1.37 (1.07, 1.74) | 1.64 (1.28, 2.12) | 1.78 (1.35, 2.34) |  |
| ≥ 28 | Reference | 1.81 (1.17, 2.78) | 1.12 (0.73, 1.72) | 1.27 (0.80, 2.01) |  |
| Central obesity |  |  |  |  | 0.1417 |
| No | Reference | 1.55 (1.09, 2.20) | 1.51 (1.04, 2.18) | 2.45 (1.66, 3.61) |  |
| Yes | Reference | 1.42 (0.95, 2.14) | 1.11 (0.74, 1.66) | 1.71 (1.09, 2.66) |  |
| Low muscle mass |  |  |  |  | 0.2940 |
| No | Reference | 1.30 (0.96, 1.74) | 1.55 (1.14, 2.10) | 1.46 (1.07, 1.98) |  |
| Yes | Reference | 1.32 (1.09, 1.60) | 1.33 (1.09, 1.62) | 1.44 (1.16, 1.77) |  |

Data are presented as odds ratio (95% confidence interval).

Models adjusted for age, region, residence area, education level, annual income, smoke history, drink history, and exercise frequency, menopausal status and BMI.

Abbreviations: BMI, body mass index.

**Table S8. Sensitivity analyses of percent regional fat and risk of** **metabolically unhealthy by sex**

| **Variables** | **Quartile 1** | **Quartile 2** | **Quartile 3** | **Quartile 4** | ***P* _for trend_** |
| --- | --- | --- | --- | --- | --- |
| **Men** |  |  |  |  |  |
| Percent arm fat |  |  |  |  |  |
| range | <6.78 | 6.78 to <7.32 | 7.32 to <7.87 | ≥7.87 |  |
| Model 1 | Reference | 1.21 (0.97, 1.50) | 1.43 (1.15, 1.79) | 0.88 (0.70, 1.11) | 0.6491 |
| Model 2 | Reference | 0.82 (0.64, 1.04) | 0.87 (0.68, 1.11) | 0.57 (0.44, 0.73) | <0.0001 |
| Percent leg fat |  |  |  |  |  |
| range | <34.15 | 34.15 to <36.00 | 36.00 to <38.52 | ≥38.52 |  |
| Model 1 | Reference | 1.00 (0.80, 1.26) | 0.68 (0.54, 0.85) | 0.30 (0.24, 0.39) | <0.0001 |
| Model 2 | Reference | 0.94 (0.74, 1.19) | 0.76 (0.60, 0.97) | 0.60 (0.46, 0.79) | <0.0001 |
| Percent trunk fat |  |  |  |  |  |
| range | <53.85 | 53.85 to <56.60 | 56.60 to <58.86 | ≥58.86 |  |
| Model 1 | Reference | 2.26 (1.79, 2.86) | 3.13 (2.47, 3.98) | 3.02 (2.36, 3.86) | <0.0001 |
| Model 2 | Reference | 1.43 (1.11, 1.84) | 1.67 (1.29, 2.17) | 1.72 (1.32, 2.24) | <0.0001 |
| **Women** |  |  |  |  |  |
| Percent arm fat |  |  |  |  |  |
| range | <7.45 | 7.45 to <8.14 | 8.14 to <8.89 | ≥8.89 |  |
| Model 1^*^ | Reference | 1.85 (1.49, 2.31) | 2.33 (1.88, 2.88) | 4.41 (3.58, 5.44) | <0.0001 |
| Model 2^*^ | Reference | 1.17 (0.93, 1.47) | 0.97 (0.75, 1.25) | 0.90 (0.65, 1.24) | 0.2498 |
| Percent leg fat |  |  |  |  |  |
| range | <35.39 | 35.39 to <38.20 | 38.20 to <41.83 | ≥41.83 |  |
| Model 1^*^ | Reference | 0.54 (0.46, 0.64) | 0.30 (0.24, 0.36) | 0.13 (0.10, 0.17) | <0.0001 |
| Model 2^*^ | Reference | 0.77 (0.63, 0.92) | 0.55 (0.43, 0.70) | 0.34 (0.25, 0.47) | <0.0001 |
| Percent trunk fat |  |  |  |  |  |
| range | <50.37 | 50.37 to <53.46 | 53.46 to <55.93 | ≥55.93 |  |
| Model 1^*^ | Reference | 2.13 (1.68, 2.69) | 3.67 (2.92, 4.61) | 5.88 (4.65, 7.43) | <0.0001 |
| Model 2^*^ | Reference | 1.41 (1.11, 1.81) | 1.91 (1.49, 2.46) | 2.34 (1.78, 3.08) | <0.0001 |

Data are presented as odds ratio (95% confidence interval).

Model 1: adjusted for age, region, residence area, education level, annual income, smoke history, drink history, and exercise

frequency.

Model 2: further adjusted for body mass index.

^*^ Models were additionally adjusted for menopausal status.

**Table S9. Correlation of percent regional fat with cardiometabolic variables by sex**

|  | Male | | |  | Female | | |
| --- | --- | --- | --- | --- | --- | --- | --- |
|  | Arm fat % | Leg fat % | Trunk fat % |  | Arm fat % | Leg fat % | Trunk fat % |
| SBP | -0.04* | -0.16*** | 0.16*** |  | 0.19*** | -0.24*** | 0.21*** |
| DBP | -0.07*** | -0.20*** | 0.19*** |  | 0.22*** | -0.30*** | 0.27*** |
| FBG | -0.04 | -0.10*** | 0.10*** |  | 0.11*** | -0.18*** | 0.17*** |
| TC | -0.09*** | -0.05** | 0.06*** |  | -0.02 | -0.09*** | 0.12*** |
| HDL | 0.00 | 0.24*** | -0.22*** |  | -0.29*** | 0.29*** | -0.25*** |
| LDL | -0.08*** | -0.07*** | 0.08*** |  | 0.03* | -0.15*** | 0.16*** |
| TG | -0.05** | -0.22*** | 0.22*** |  | 0.23*** | -0.33*** | 0.31*** |

Results represent Spearman correlation coefficients adjusted for age.

Abbreviations: SBP, systolic blood pressure; DBP, diastolic blood pressure; FBG, fasting blood glucose; TC, total cholesterol; HDL-C, high-density lipoprotein cholesterol; LDL-C, low-density lipoprotein cholesterol; TG, triglycerides.

⁎ p ≤ 0.05; ⁎⁎ p ≤ 0.01; ⁎⁎⁎ p ≤ 0.001.

**
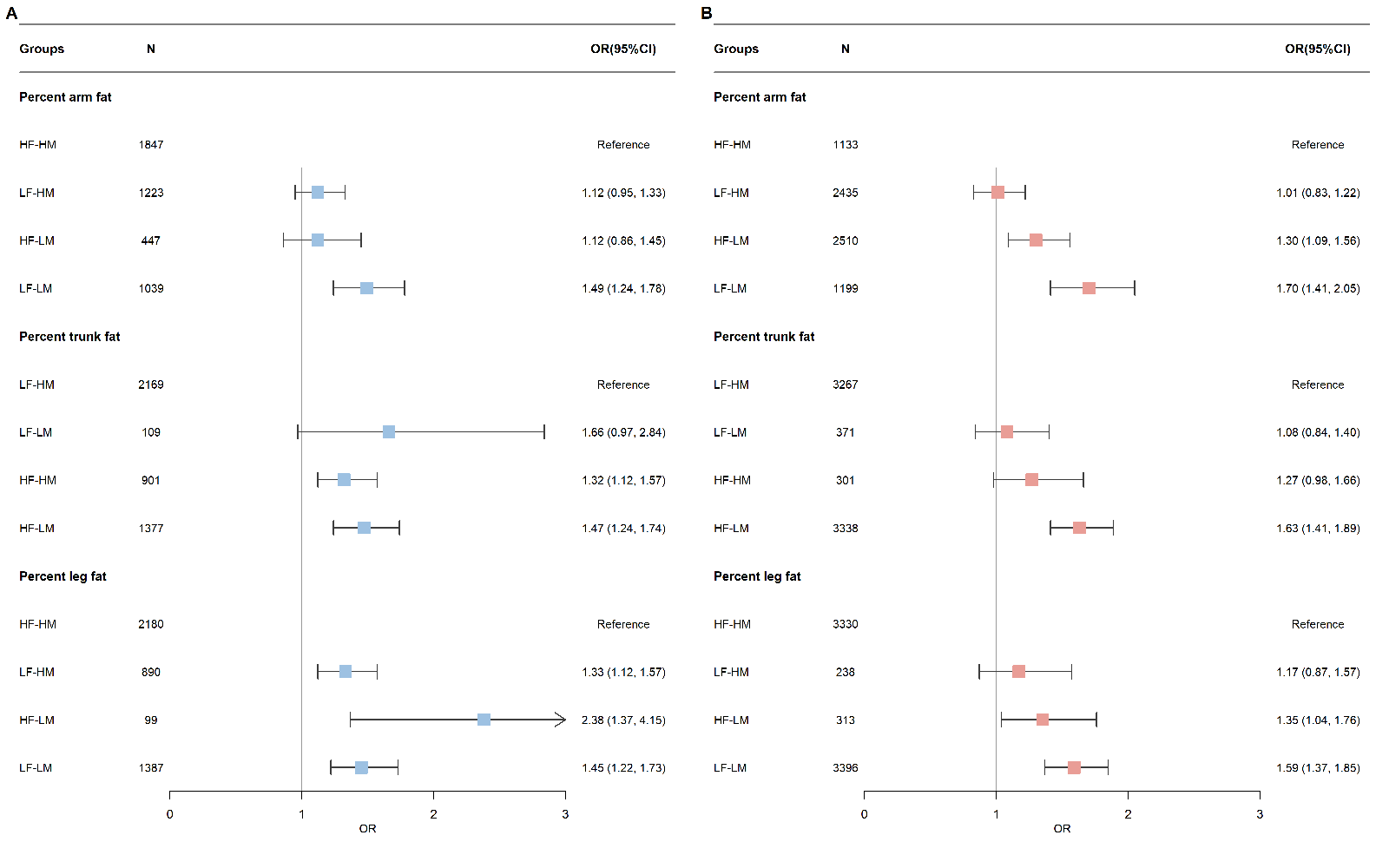
**

**Figure S1. Adjusted odds ratios with 95% confidence intervals for metabolically unhealthy by body composition group (A for men; B for women).** LF-LM, low fat-low muscle group; LF-HM, low fat-high muscle group; HF-LM, high fat-low muscle group; HF-HM, high fat-high muscle group. We subdivided men and women into four groups based on regional fat mass and appendicular skeletal muscle mass index (ASMI). Regional fat mass was categorized as low or high using the median value as the cutoff. Low muscle mass was defined as an ASMI of less than 33.0% in men and 28.0% in women; values above these thresholds were classified as high muscle mass. Models were adjusted for age, region, residence area, education level, annual income, smoke history, drink history, exercise frequency, and body mass index. Models for women were additionally adjusted for menopausal status.
